# Associations between the time of conception and the shape of the lactation curve in early lactation in Norwegian dairy cattle

**DOI:** 10.1186/1751-0147-53-5

**Published:** 2011-02-08

**Authors:** Fredrik Andersen, Olav Østerås, Olav Reksen, Nils Toft, Yrjo T Gröhn

**Affiliations:** 1 Department of Production Animal Clinical Sciences, Norwegian School of Veterinary Science, Oslo, Norway; 2 Department of Large Animal Sciences, Faculty of Life Sciences, University of Copenhagen, Copenhagen, Denmark; 3 Department of Population Medicine and Diagnostic Sciences, College of Veterinary Medicine, Cornell University, Ithaca, New York, USA

## Abstract

**Background:**

This study was carried out to determine if an association exists between the shape of the lactation curve before it is influenced by the event of conception and the time from calving to conception in Norwegian dairy cattle. Lactation curves of Norwegian Red cows during 5 to 42 days in milk (DIM) were compared between cows conceiving between 43 and 93 DIM and cows conceiving after 93 DIM.

**Methods:**

Data from 23,049 cows, represented by one lactation each, with 219,538 monthly test days were extracted from the Norwegian Dairy Herd Recording System, which represents 97% of all Norwegian dairy cows. Besides veterinary treatments, these records also included information on daily milk yield at monthly test days. The data were stratified by parity groups (1, 2, and 3 and higher) and time to conception periods (43-93 DIM and >93 DIM). The sample was selected using the following selection criteria: conception later than 42 DIM, calving season July to September, no records of veterinary treatment and the level of energy fed as concentrates between 8.69 and 12.83 MJ. The shape of the lactation curves were parameterized using a modified Wilmink-model in a mixed model analysis. Differences in the parameters of the lactation curves with different conception times were evaluated using confidence intervals.

**Results:**

Lactation curves characterized by a low intercept and a steep ascending slope and a steep descending slope were associated with early conception across all parities. The peak milk yield was not associated with time of conception.

**Conclusions:**

A practical application of the study results is the use of the shape of the lactation curve in future herd management. Groups of cows with impaired reproductive performance may be identified due to an unfavorable shape of the lactation curve. Monitoring lactation curves and adjusting the feeding strategy to adjust yield therefore may be useful for the improvement of reproductive performance at herd level.

## Background

Good reproductive performance is a key element in the modern dairy industry. Norwegian Red is the most common dairy breed in Norway, constituting 94% of the Norwegian dairy cow population and is kept for both milk and beef production [1]. A recent study using 829 animals to investigate the reproductive performance of this breed, reported the following fertility measures after the first artificial insemination. An overall 60-d nonreturn rate of 72.5%, an overall pregnancy incidence of 63.8% and an overall calving rate of 57.2%. Even if these numbers indicate good reproductive performance of the breed, there is some variation in calving to first insemination, 85.3 days (SD ± 41.9) [2]. Management, such as estrus detection and feeding strategy, is of major importance for the reproductive performance in a dairy herd [3,4]. Management strategies can be difficult to measure and compare in a large scale epidemiological study. The shape of the lactation curve, however, may reflect the feeding strategy in an objective manner [5]. Feeding during the transition period and the upscaling of concentrates fed after calving has been shown to be of importance to the energy balance [3,5,6]. Because cows do not conceive as long as their energy balance is decreasing [7], the link between feeding, energy balance and the shape of the lactation curve is also expected to be associated with the time from calving to conception [8].

The calving to conception period is commonly used as a measure of reproductive performance. It consists of the period from calving to first service and the period from first service to conception in cows not conceiving at first service. A prolonged calving to conception period results in an extended lactation period which has been shown to give a higher total milk yield in the current lactation [9,10]. Even if a long calving to conception period is beneficial in terms of the magnitude of the milk yield in the current lactation, it is still regarded to be a measure of impaired reproductive performance. In terms of milk production, lifetime production is more important than production in individual lactations [11].

The Norwegian cattle breeding organization, Geno, recommends not to start breeding before six weeks after calving. Further, the annual statistics from the Norwegian Dairy Herd Recording System (NDHRS) gives a mean calving to first artificial insemination (AI) time of 86.9 days. This database includes 97% of all Norwegian dairy cows [1].

The lactation curve in dairy cattle describes the pattern of milk yield throughout the lactation period. The shape of this curve and the factors influencing it have been investigated in several studies [12,13]. To find the association between a reproductive trait and the shape of the lactation curve, the latter needs to be parameterized. Earlier studies have shown that there are several methods available to obtain an estimated lactation curve with good fit to observed data using monthly milk yield records [14-17].

Observing the shape of the lactation curve may be helpful in differentiating factors related to feeding or estrus detection, such as shifting the focus of management interventions to estrus detection if the shape of the lactation curve is considered optimal or vice versa. The aim of this study was to determine whether an association exists between the shape of the lactation curve before it is influenced by the event of conception and the time from calving to conception in Norwegian dairy cattle. The rationale is that there is a relationship between feeding and the shape of the lactation curve. Adjusting the feeding strategy in the first part of lactation may thus optimize the shape of the lactation curve and thereby shorten the time to conception.

## Methods

### Material

Data used in this study were extracted from the NDHRS, which is a single database containing production data, veterinary diagnoses, information on AI and treatments [1]. Daily milk yield and daily amount of concentrates fed are recorded on monthly test days and the test day record also states if the cow was diseased when daily milk yield was measured. The database provides yearly statistics of disease incidences, feeding and production at herd level. The validity of this database is considered high because Norwegian legislation prohibits farmers from using veterinary drugs and requires veterinarians to register all diagnoses and treatments [18].

Sampling from the database was conducted to extract data on dairy cows starting their lactation on or after January 1^st^, 2005 and ending their lactation no later than February 19^th^, 2007. Only the first complete lactation from each cow in the period was used and all breeds other than Norwegian Red were excluded. All lactations started and ended with a successful calving and the date of the last AI was used to determine the day of conception. Lactations with a gestation length of less than 269 days or more than 289 days were considered false recordings and deleted (5.0%). Lactations with fewer than 4 monthly milk yield records were deleted (4.1%). If the cow was reported as diseased on test day, if the test day occurred before 5 DIM or if 0 kg milk was recorded on test day, the daily milk yield record was deleted (4.7%).

A study population was selected from the extracted data, including only lactations without records of veterinary treatments (68.31%). From this study population a study sample was selected using the following criteria. Lactations where the first date of AI occurred before the recommended 42 DIM were deleted (4.28%). Lactations with calving recorded from July to September (35.78%), and a mean level of daily energy fed as concentrates between 8.69-12.83 MJ, calculated from amount of concentrates given at each test day (63.39%), were selected for the study.

The data were stratified into parity groups: 1 = first parity, 2 = second parity and 3 = third or later parities, and early conceivers, TIME_CC = 1 (43-93 DIM) and late conceivers, TIME_CC = 2 (> 93 DIM). The final study sample used for the statistical analysis consisted of 219,538 test days in 23,049 lactations (Table 1).

**Table 1:** Descriptive statistics within parity and time to conception of study sample with calving season July to September, average of 8.69 MJ to 12.83 MJ of daily energy from concentrates and no records of veterinary treatments throughout lactation, of Norwegian dairy cattle during the period 2005 to 2007.

	N lactations	Mean age at calving in months (95%CI)	Lactations with early conception (43 to 93 DIM)			Lactations with late conception (after 93 DIM)		
			
			N lactations	Calving to first insemination in days (95%CI)	Number of inseminations	N lactations	Calving to first insemination in days (95%CI)	Number of inseminations
First parity lactations	12,758	25.48 (25.42-25.53)	6,883	69.84 (69.51-70.17)	1.27 (1.25-1.28)	5,875	97.21 (96.49-97.92)	2.12 (2.10-2.15)
Second parity lactations	5,651	37.63 (37.54-37.73)	3,131	70.23 (69.73-70.72)	1.25 (1.23-1.26)	2,520	98.01 (96.89-99.12)	2.02 (1.98-2.06)
Third or later parity lactations	4,640	58.40^1^	2,574	70.99 (70.46-71.52)	1.25 (1.23-1.27)	2,066	97.94 (96.69-99.18)	2.00 (1.96-2.05)
All lactations	23,049	35.10^1^	12,588	70.17 (69.93-70.42)	1.26 (1.25-1.27)	10,461	97.54 (97.00-98.09)	2.08 (2.05-2.10)

^1 ^95%CI not applicable

### Study Design and Method

The study was performed as a retrospective cohort study with a closed population at lactation level. To minimize the effect of year, calving season and breed differences the described selection criteria were used to generate the study sample. A modified Wilmink model [16] was used to estimate the shape of the lactation curve adjusted for the effects of time to conception and parity.

A mixed model was run using PROC MIXED (SAS Institute Inc., 2003) with repeated measurements of test day milk yields within lactation nested within herd to account for the cluster effect. A spatial power correlation matrix, SP(POW), with month as time scale was chosen for the repeated statement. The SP(POW) correlation matrix allowed for non-equidistant timepoints and was chosen after evaluating different relevant matrices using the Akaike Information Criterion. Further, the model was run with random regression of the variable describing the natural logarithm of DIM (lnDIM) at herd level to account for herd level variation. A backward selection process with inclusion criteria of P < 0.05 based on the F-test was used to build the final model. Significant two-way interaction terms were included in the final model.

**Figure 1:**
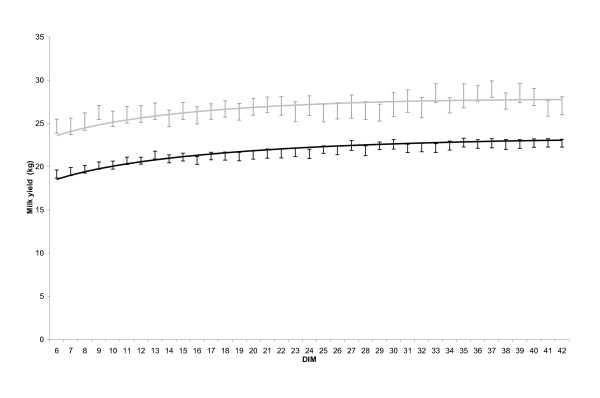
**Estimated lactation curves from 5 to 42 DIM (▬▬) compared with 95% CI of raw data milk yields of 12,758 first parity lactations (I) and estimated lactation curves (▬▬) compared with 95%CI of raw data milk yields of 5,651 second parity lactations (I) with calving season July to September, average of 8.69 MJ to 12.83 MJ of daily energy from concentrates and no records of veterinary treatments throughout lactation, of Norwegian dairy cattle during the period 2005 to 2007**.

Equation I:

$$\begin{array}{l} {\text{Y}}_{\text{ijk}}=\beta_{0}+\beta_{1}\ln{\text{DIM}}_{\text{ijk}}+\beta_{2}{\text{DIM}}_{\text{ijk}}+\beta_{3}{\text{TIME\_CC}}_{\text{jk}}+\beta_{4}{\text{PAR}}_{\text{jk}}+\beta_{5}\ln{\text{DIM}}_{\text{ijk}}{\text{TIME\_CC}}_{\text{jk}}+\\ \beta_{6}\ln{\text{DIM}}_{\text{ijk}}{\text{PAR}}_{\text{jk}}+\beta_{7}{\text{DIM}}_{\text{ijk}}{\text{TIME\_CC}}_{\text{jk}}+\beta_{8}{\text{DIM}}_{\text{ijk}}{\text{PAR}}_{\text{jk}}+\varepsilon_{\text{ijk}} \end{array}$$

where subscript ijk identifies the i-th test day in the j-th lactation in the k-th herd. Y is milk yield (kg), DIM is the number of days from calving to the test-day, lnDIM is the natural logarithm of DIM, TIME_CC is the conception day group, PAR is the parity group and ε is the error term. The β-values are associated with the starting level of milk production (kg) (β_0_), the ascending slope of the lactation curve (N(β_1_,σ^2^_β1_) i.e. modeled as a random slope), the descending slope of the lactation curve (β_2_), the interaction effect of TIME_CC with the starting level of milk production (β_3_), the interaction effect of PAR with the starting level of milk production (β_4_), the interaction effect of TIME_CC with the ascending slope (β_5_), the interaction effect of PAR with the ascending slope (β_6_), the interaction effect of TIME_CC with the descending slope (β_7_) and the interaction effect of PAR with the descending slope (β_8_). Residuals (ε_ijk _) were modeled using the SP(POW) structure as previously mentioned. The underlying assumptions of the models were assessed visually by QQ-plots testing the normality of the residuals, and predicted values were plotted against residuals to assess the homogeneity of variance of the residuals. The fit of the estimated lactation curves were tested by plotting them against the raw milk records and their 95% confidence interval (Figure 1).

**Figure 2:**
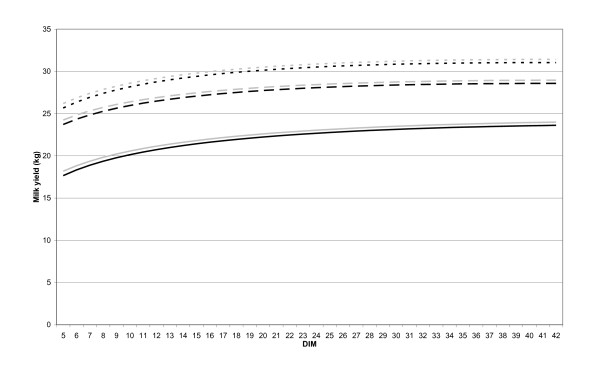
**Lactation curves from 5 to 42 DIM of lactations with calving season July to September, average of 8.69 MJ to 12.53 MJ of daily energy from concentrates and no records of veterinary treatments throughout lactation, of Norwegian dairy cattle during the period 2005 to 2007**. 6,883 first parity lactations with early conception (43 to 93 DIM) (▬▬), 5,875 first parity lactations with late conception (after 93 DIM) (▬▬), 3,131 second parity lactations with early conception (43 to 93 DIM) (▬ ▬), 2,520 second parity lactations with late conception (after 93 DIM) (▬ ▬), 2,574 third or later parity lactations with early conception (43 to 93 DIM) (▪ ▪ ▪) and 2,006 third or later parity lactations with late conception (after 93 DIM) (▪ ▪ ▪).

The estimates from the models were used to generate different lactation curves of early and late conceivers (Figure 2). These values and their standard errors were also used directly as parameters for the milk yield at the onset of lactation (β_0_), the ascending slope (β_1_) and descending slope (β_2_), which were compared between TIME_CC classes using confidence intervals (Table 2). Finally the β-values were used for calculation of peak milk yield (-β_1_/β_2_) and the day of peak milk yield (β_0_+(β_1_×ln(peak milk yield))+ (β_2_×peak milk yield)).

**Table 2:** Lactation curve parameters of lactations with calving season July to September, average of 8.69 MJ to 12.83 MJ of daily energy from concentrates and no records of veterinary treatments throughout lactation, of Norwegian dairy cattle during the period 2005 to 2007.

	Early conception (from 43 to 93 DIM)		Late conception (after 93 DIM)	
	
	Estimate	SE^4^	Estimate	SE^4^
**First parity (12,758 lactations and 121,552 test days)**				
Intercept (kg)	12.83***	0.11	13.94***	0.11
Ascending slope (kg/days)	3.42***	0.03	2.96***	0.03
Descending slope (kg/days)	-0.0643***	0.0004	-0.0519***	0.0004
Peak day^1^	53.19 (51.57-54.85)^3^**		57.13 (55.05-59.27) ^3^**	
Peak yield^2 ^(kg)	23.01 (22.51-23.51) ^3^		22.96 (22.43-23.50) ^3^	
**Second parity (5,651 lactations and 53,779 test days)**				
Intercept (kg)	19.13***	0.14	20.23***	0.15
Ascending slope (kg/days)	3.17***	0.04	2.71***	0.04
Descending slope (kg/days)	-0.0814***	0.0005	-0.0689***	0.0005
Peak day^1^	38.95 (37.45-40.48) ^3^		39.33 (37.51-41.21) ^3^	
Peak yield^2 ^(kg)	27.57 (26.95-28.19) ^3^		27.48 (26.83-28.13) ^3^	
**Third or later parity (4,640 lactations and 44,207 test days)**				
Intercept (kg)	19.81***	0.15	20.91***	0.16
Ascending slope (kg/days)	3.89***	0.05	3.43***	0.05
Descending slope (kg/days)	-0.0984***	0.0006	-0.0859***	0.0006
Peak day^1^	39.53 (38.18-40.90) ^3^		39.92 (38.35-41.54) ^3^	
Peak yield^2 ^(kg)	30.22 (29.55-30.89) ^3^		30.13 (29.44-30.82) ^3^	

** estimates within same row differ (p < 0.05) *** estimates within same row differ (p < 0.001)

^1 ^- ascending slope/descending slope

^2 ^starting milk yield + (ascending slope×ln(peak day)) + (descending slope×peak day)

^3 ^interval calculated from 95% lower and upper confidence limits of estimates

^4 ^Standard Error

## Results

Descriptive statistics of the study sample, with calving season July to September and 305-d lactation mean level of daily energy fed as concentrates between 8.69 MJ and 12.83 MJ, are given in Table 1. The herds in the study population had an average size of 18.7 cow-years and an average milk yield per lactation of 6,665 kg milk. Cow-years were defined as the sum of the number of cows on each day on a farm divided by 365 days, reflecting the mean herd size during one year. Component feeding was commonly used which on average at farm level consisted of 42.0% concentrates, 41.4% grass silage, 14.2% pasture and 2.4% other feedstuffs. Differences in age at calving within each parity were assumed to be influential a priori, but were found to be non-significant. The lactations with early conception were inseminated significantly earlier and significantly fewer times compared to the lactations conceiving late, both in total and within parity groups (Table 1). The fit of the lactation curve generated from the parameters estimated in the model was compared visually with the 95%CI of raw data test day milk yields (Figure 1).

The parameters describing the lactation curve of early conceivers (43-93 DIM) were significantly different compared to the parameters describing the lactation curve of late conceivers (after 93 DIM). Cows conceiving early had significant lower intercept, steeper ascending slope and steeper descending slope compared to cows conceiving late, p < 0.001 (Figure 2 and Table 2). These results were the same across all parity groups. Peak yield as well as peak day, did not differ significantly with conception day groups for any parity group, except for peak day in first parity (Table 2).

The covariance estimates were 0.99 for the correlation matrix and 0.22 for the random regression of lnDIM, which gave intraclass correlation coefficients of 0.058 and 0.013 respectively. This means that repeated measurements and parameter lnDIM contribute 5.8% and 1.3% respectively, to the between herd variation of the model parameters.

## Discussion

In the current study a lactation curve described by a low starting milk yield and a steep slope was associated with early conception across all parities. This concurs with an earlier investigation which found that high early milk yield was associated with a longer period of days open [10]. The present study indicate that this difference is observable before 15 DIM (Figure 2). A too high starting milk yield would presumably maintain the NEB and thereby prolong the time to conception [7]. An increased number of A.I. per conception indicate impaired reproduction (Table 1). The material used, however, could not answer if delayed breeding start is voluntary or a result of silent heat.

The peak milk yield seemed to have no association with the time of conception. Higher milk yield has been associated with longer time to resumption of ovarian activity in high yielding dairy cows in some reports [8,19,20]. Other authors have found no [21,22] or inverse associations between milk yield and recurrence of ovarian activity [23,24].

In the current study a steep ascending slope early in lactation is associated with early resumption of breeding activity. The biological reason for this might be the found in energy coverage. Earlier studies have found that the change in body condition score and the magnitude of the negative energy balance (NEB) in early lactation are crucial to conception [25,26]. A steep ascending slope of the lactation curve may be indicative of adequate energy coverage during the early post partum period and thereby early resumption of ovarian activity, found in the current study. An earlier Norwegian study found that cows experiencing early versus delayed resumption of ovarian activity have lactation curves with different slopes as energy balance approaches zero throughout the post partum period [27]. A risk of choosing a feeding strategy based on a high escalation rate of concentrates to ensure adequate energy coverage is the increased risk of indigestion because of too sudden changes in the ruminal environment. Cows of the current study that succeeded in becoming pregnant appeared to have counteracted this negative effect of concentrate as indigestion is likely to lead to lower milk yield and impaired reproduction [28,29].

A confounding variable not taken into account in the current study, because of the lack of data, is the voluntary waiting period (VWP), which is the time period decided by the farmer before breeding starts. Although the average 305 d yield in the current study was less than 7000 kg, one cannot rule out that a high milk yield early in lactation, might have lead farmers to prolong the VWP to avoid the problems related to drying of in some high yielding cows. On the other hand, a high yielding cow might be looked upon by the farmer as hard to get pregnant and therefore started to be bred earlier i.e. a shorter VWP, which is supported by a high number of A.I in late conceiving cows (Table 1). In other words, management decisions based on high yield early in lactation, consistent with a steep ascending slope, might influence the calving to conception time in either way. However, the principal aim of this study was to investigate whether there is an association between the lactation curve and time to conception in Norwegian dairy cows. The reason for delayed conception may be biological, or managerial, or as is more often the case, a combination of both. Nevertheless, the alteration in the profile of the lactation curve may pose an opportunity of early identification of a herd reproductive problem that reaches beyond estrus detection and A.I. management. When the problems are to be ruled out at the herd level by trained personnel, additional information on voluntary wait, culling policy and feeding management will probably be readily obtainable from the herdsmen.

The current study identified an association between a steep descending slope of the lactation curve and early conception. The reason behind this association might be the negative effect pregnancy has on milk yield [30,31]. This effect and its magnitude on milk yield is beyond the scope of this paper and we hope to be able to pursue this question in future research programs.

In the current study the lactation curves were estimated by using a modified Wilmink model and the statistical setup of a mixed model using monthly test day records of daily milk yield. A concern with the current method may be that milk records obtained after conception might influence the estimated shape of the curve before conception. The fit of the curves generated from using all milk records to raw data proved to be satisfying (Figure 1). Potential confounding of correlation between test-day milk yields and clustering within lactation was taken care of by running the model with repeated measurements of test day milk yield within lactation nested within herd.

Another concern related to the association between conception time and the shape of the lactation curve is culling of animals because of reproductive failure. The farmer's decision whether to give up on getting a cow pregnant may be influenced by the course of the lactation curve. Cows with a less persistent lactation curve, i.e. a steep descending slope of the lactation curve, may be given up earlier than other cows. This would cause a problem if the primary aim of the study was to assess reproductive performance at herd level. The following reasoning applies to the selection criteria used in the current study where non-pregnant cows and culled cows, 30.2% of the observations, were excluded. The most prevalent reasons for culling are low milk yield, reproductive failure, disease and age [29]. Cows omitted from the study for non-pregnancy are likely to have followed the lactation curve pattern of late conceivers with a high persistency of the lactation curve [30,31]. Including these cows would probably have emphasized the difference in lactation curve traits between late and early conceiving cows. There might be a risk however, that the late conceivers are overrepresented among high performing cows. The reason for this being the increased likelihood of culling a low performing cow compared to a high performing cow. In the material used in the current study, peak milk yield was virtually equal across calving to conception intervals within each parity category (Table 2), and therefore this bias of performance is believed to be minimal.

Disease is known to have an effect on both milk production [28] and reproductive performance [29]. To avoid the influence of disease on the association, only data from lactations with no records of veterinary treatments (68.3%) were used in the current study. The data used in the current study were obtained from the NDHRS database, which has not been validated yet. A major and currently ongoing Nordic research project will validate all the Nordic dairy health recording systems. There might be problems with non-directional misclassification and measurement errors which weaken the statistical power of the result using non-validated sources. Research has shown, however, that adjusting the sample size can account for this potential loss of power [32] and this is well taken care of by the large sample size used in the current study.

The strength of the current study, using a large representative database and a large sample size, is that the results can be implemented on the study population of Norwegian dairy cattle. One must consider, however, that we have restricted our analysis to certain calving season July to September. The reason for this approach is that internal validity is more important than external validity. We also, however, tested this for different calving seasons and found the same association. Therefore we are confident that an association between the time of conception and the shape of the lactation curve does indeed exist. Plotting daily milk records obtained from the automatic milking system, the shape of the lactation curve might be monitored, adjusted by feeding and optimized for conception. Together with heat detection and insemination technique this is yet another tool to manage the reproductive performance in the dairy herd.

## Conclusions

An earlier Norwegian study indicates that there is a dynamic relationship between NEB, milk yield and reproductive performance in dairy cows [27]. The current study results point to the same hypothesis. Starting the lactation with high yield and slightly flatter slope is associated with later conception, despite having the same peak milk yield. A smaller scale controlled clinical trial is required to evaluate possible causation of this association.

## Competing interests

The authors declare that they have no competing interests.

## Authors' contributions

Study design: FA, OØ, OR and YTG. Data collection: FA and OØ. Data analysis: FA and OØ. Statistical analysis: FA, OØ and NT. Writing paper: FA. Critical review and approval of the final manuscript: all authors.

## References

[B1] Østerås O Solbu H Refsdal AO Roalkvam T Filseth O Minsaas A Results and evaluation of thirty years of health recordings in the Norwegian dairy cattle population J Dairy Sci 2007 90 4483 4497 17699070 10.3168/jds.2007-0030

[B2] Garmo RT Refsdal AO Kalberg K Ropstad E Waldmann A Beckers JF Reksen O Pregnancy incidence in Norwegian Red Cows using nonreturn to estrus, rectal palpation, pregnancy associated glycoproteins and progesterone J Dairy Sci 2008 91 3025 3033 10.3168/jds.2007-0778 18650279

[B3] Spiekers H Klunter AM Potthast V Pfeffer E Effects of different concentrate levels on milk yield, feed intake, live weight change, health and reproduction in dairy cows Livest Prod Sci 1991 28 89 105 10.1016/0301-6226(91)90001-7

[B4] Roche JR Friggens NC Kay JK Fisher MW Stafford JK Berry PD Invited review: Body condition score and its association with dairy cow productivity, health, and welfare J Dairy Sci 2009 92 5769 5801 10.3168/jds.2009-2431 19923585

[B5] Yrjanen S Kaustell K Kangasniemi R Sariola J Khalili H Effects of concentrate feeding strategy on the performance of dairy cows housed in a free stall barn Livest Prod Sci 2003 81 173 181 10.1016/S0301-6226(02)00276-2

[B6] Beever DE The impact of controlled nutrition during dry period on dairy cow health, fertility and performance Anim Reprod Sci 2006 96 212 226 10.1016/j.anireprosci.2006.08.002 16949220

[B7] Ferreira AM Sá WF Viena JHM Camargo LSA Pereira PAC Fernandes CAC Feed intake restriction, conception rate and parturition to conception interval in crossbred Gir-Holstein cows Anim Reprod 2005 2 135 138

[B8] Beam SW Butler WR Energy balance, metabolic hormones, and early postpartum follicular development in dairy cows fed prilled lipid J Dairy Sci 1998 81 121 131 10.3168/jds.S0022-0302(98)75559-6 9493087

[B9] Oltenacu PA Rounsaville TR Milligan RA Hintz RL Relationship between days open and cumulative milk yield at various intervals from parturition for high and low producing cows J Dairy Sci 1980 63 1317 1327 10.3168/jds.S0022-0302(80)83083-9

[B10] Lee JK VanRaden PM Norman HD Wiggans GR Meinert TR Relationship of yield during early lactation and days open during current lactation with 305-day yield J Dairy Sci 1997 80 771 776 10.3168/jds.S0022-0302(97)75997-6 9149972

[B11] Togashi K Lin CY Genetic improvement of total milk yield and total lactation persistency of the first three lactations in dairy cattle J Dairy Sci 2008 91 2836 2843 10.3168/jds.2007-0783 18565941

[B12] Tekerli M Akinci Z Dogan I Akcan A Factors affecting the shape of lactation curves of Holstein cows from the Baliksir province of Turkey J Dairy Sci 2000 83 1381 1386 10.3168/jds.S0022-0302(00)75006-5 10877405

[B13] Hansen JV Friggens NC Hojsgaard S The influence of breed and parity on milk yield, and milk yield acceleration curves Livest Sci 2006 104 53 62 10.1016/j.livsci.2006.03.007

[B14] Wood PDP A simple model of lactation curves for milk yield, food requirement and body weight Nature 1979 28 55 63

[B15] Rowlands GJ Lucey S Russell AM A comparison of different models of the lactation curve in daily cattle Anim Prod 1982 35 135 144 10.1017/S0003356100000908

[B16] Wilmink JBM Comparison of different methods of predicting 305-day milk yield using means calculated from within-herd lactation curves Livest Prod Sci 1987 17 1 17 10.1016/0301-6226(87)90049-2

[B17] Macciotta NPP Vicario D Cappio-Borlino A Detection of different shapes of lactation curve for milk yield in dairy cattle by empirical mathematical models J Dairy Sci 2005 88 1178 1191 10.3168/jds.S0022-0302(05)72784-3 15738251

[B18] Ministry of Agriculture and Food Regulations related to marking, recording and reporting of animals 2002 http://www.lovdata.no/cgi-wift/ldles?doc=/sf/sf/sf-20020903-0970.html full text of regulation is given in Norwegian

[B19] Stevenson JS Britt JH Relationships among luteinizing hormone, estradiol, progesterone, glucocorticoids, milk yield, body weight and postpartum ovarian activity in Holstein cows J Anim Sci 1979 48 570 577 528417 10.2527/jas1979.483570x

[B20] Nebel RL McGillard ML Interactions of high milk yield and reproductive performance in dairy cattle J Dairy Sci 1993 76 3257 3268 10.3168/jds.S0022-0302(93)77662-6 8227645

[B21] Villa-Godoy A Hughes TL Emery RS Chapin LT Fogwell RL Association between energy balance and luteal function in lactation dairy cows J Dairy Sci 1988 71 1063 1072 10.3168/jds.S0022-0302(88)79653-8 3392301

[B22] Harrison RO Ford SP Young JW Conley AJ Freeman AE Physiology and management: Increased milk production versus reproductive and energy status of high yielding dairy cows J Dairy Sci 1990 73 2749 2758 10.3168/jds.S0022-0302(90)78960-6 2283405

[B23] Staples CR Thacher WW Relationship between ovarian activity and energy status during the early postpartum period of high producing dairy cows J Dairy Sci 1990 73 938 947 10.3168/jds.S0022-0302(90)78750-4 2345204

[B24] Lucy MC Savio JD Badinga L De La Sota RL Thacher WW Factors that affect ovarian follicular dynamics in cattle J Anim Sci 1992 70 3615 3626 1459922 10.2527/1992.70113615x

[B25] Shrestha HK Nakao T Suzuki T Akita M Higaki T Relationships between body condition score, body weight, and some nutritional parameters in plasma and resumption of ovarian cyclicity postpartum during pre-service period in high-producing dairy cows in a subtropical region in Japan Theriogenology 2005 64 855 866 10.1016/j.theriogenology.2004.12.007 16054491

[B26] Roche JF The effect of nutritional management of the dairy cow on reproductive efficiency Anim Repro Sci 2006 96 282 296 10.1016/j.anireprosci.2006.08.007 16996705

[B27] Reksen O Gröhn YT Havrevoll OS Bolstad T Waldmann A Ropstad E Influence of concentrate allocation and energy balance on postpartum ovarian activity in Norwegian cattle J Dairy Sci 2001 84 1060 1068 10.3168/jds.S0022-0302(01)74566-3 11384032

[B28] Fourichon C Seegers H Bareille N Beaudeau F Effects of disease on milk production in the dairy cow: a review Prev Vet Med 1999 41 1 35 10.1016/S0167-5877(99)00035-5 10416197

[B29] Gröhn YT Rajala-Schultz PJ Epidemiology of reproductive performance in dairy cows Anim Repro Sci 2000 60-61 605 614 10.1016/s0378-4320(00)00085-3 10844228

[B30] Olori VE Brotherstone S Hill WG McGuirk BJ Effect of gestation stage on milk yield and composition in Holstein Friesian dairy cattle Livest Prod Sci 1997 52 167 176 10.1016/S0301-6226(97)00126-7

[B31] Brotherstone S Thompson R White IMS Effects of pregnancy on daily milk yield of Holstein-Friesian dairy cattle Livest Prod Sci 2004 87 265 269 10.1016/j.livprodsci.2003.07.014

[B32] Divine OJ Smith JM Estimating sample size for epidemiologic studies: the impact of ignoring exposure measurement uncertainty Statistics in Medicine 1998 17 1375 1389 10.1002/(SICI)1097-0258(19980630)17:12<1375::AID-SIM857>3.0.CO;2-D 9682326

